# The Effect of the Modification of Carbon Spheres with ZnCl_2_ on the Adsorption Properties towards CO_2_

**DOI:** 10.3390/molecules27041387

**Published:** 2022-02-18

**Authors:** Iwona Pełech, Piotr Staciwa, Daniel Sibera, Ewelina Kusiak-Nejman, Antoni W. Morawski, Joanna Kapica-Kozar, Urszula Narkiewicz

**Affiliations:** 1 Department of Inorganic Chemical Technology and Environment Engineering, Faculty of Chemical Technology and Engineering, West Pomeranian University of Technology in Szczecin, Pułaskiego 10, 70-322 Szczecin, Poland; ipelech@zut.edu.pl (I.P.); daniel.sibera@zut.edu.pl (D.S.); ekusiak@zut.edu.pl (E.K.-N.); antoni.morawski@zut.edu.pl (A.W.M.); joanna.kapica@zut.edu.pl (J.K.-K.); urszula.narkiewicz@zut.edu.pl (U.N.); 2 Department of General Civil Engineering, Faculty of Civil and Environmental Engineering, West Pomeranian University of Technology in Szczecin, Piastów 50a, 70-311 Szczecin, Poland

**Keywords:** carbon spheres, carbon dioxide, adsorption, microwave reactor, zinc oxide, zinc chloride

## Abstract

Zinc chloride and potassium oxalate are often applied as activating agents for carbon materials. In this work, we present the preparation of ZnO/carbon spheres composites using resorcinol-formaldehyde resin as a carbon source in a solvothermal reactor heated with microwaves. Zinc chloride as a zinc oxide source and potassium oxalate as an activating agent were applied. The effect of their addition and preparation conditions on the adsorption properties towards carbon dioxide at 0 °C and 25 °C were investigated. Additionally, for all tested sorbents, the CO_2_ sorption tests at 40 °C, carried out utilizing a thermobalance, confirmed the trend of sorption capacity measured at 0 and 25 °C. Furthermore, the sample activated using potassium oxalate and modified using zinc chloride (a carbon-to-zinc ratio equal to 10:1) displayed not only a high CO_2_ adsorption capacity (2.69 mmol CO_2_/g at 40 °C) but also exhibited a stable performance during the consecutive multicycle adsorption–desorption process.

## 1. Introduction

The greenhouse effect results from the emission into the atmosphere of different chemical compounds, among them water vapor, carbon dioxide, methane, ozone and CFCs (freons). These gases may come from natural sources and can be produced due to the human activity related to the development of the energy industry, progressive urbanization and the burning of fossil fuels, such as coal, oil or natural gas. As a result of these actions, the increase in the planet’s temperature, called global warming, has been observed, affecting species extinction, changes in the Earth’s hydrologic cycle, and contributing to climate anomalies. Gases able to trap heat in the atmosphere are called greenhouse gases. The primary greenhouse gas is carbon dioxide. Recently, CO_2_ has been removed from gas mixtures through absorption in liquids, as amine or potassium carbonate solutions. Unfortunately, these technologies are highly energy consuming and not environmentally friendly. The removal of CO_2_ can also be achieved by natural solutions, such as tree planting or new technologies for gas capture and storage in geological formations (CCS) [1,2]. Much more prospective technologies are post-combustion CO_2_ capture and utilization (CCU), enabling the transformation of carbon dioxide into useful products (fuels or other chemicals) [3].

Apart from activated carbons [4], ordered porous carbons [5], activated carbon fibers [6] or graphene [7], carbon spheres seem to be an excellent candidate for carbon dioxide capture. They are characterized by unique chemical and physical properties: a high specific surface area, large pore volume, well-defined pore size distribution, chemical stability, low cost and the possibility of modification with heteroatoms. The synthesis of carbon spheres is based on the high temperature decomposition of carbon-containing materials in an inert atmosphere (chemical vapor deposition, arc-discharge and laser ablation) or the low-temperature pyrolysis and catalytic decomposition of organic compounds by the heat treatment of polymers or other materials, often in an autoclave [8]. Instead of an autoclave, a microwave reactor can be successfully used, which allows for a significant reduction in the processing time of up to several minutes [9,10,11]. The adsorption properties of the obtained materials depend on the experimental conditions, e.g. carbonization temperature, activation step and carbon source. Due to the very low content of impurities and ease of modification as precursors for carbon materials, polymers are widely used [12,13], used are resins based on phenol [14], urea [15], melamine [16] or resorcinol. The conversion of organic matters into carbon materials usually occurs at elevated temperatures and is performed at atmospheric pressure under an inert gas flow [10,17]. To develop the surface area and improve the adsorption capacity of carbon materials, physical and chemical activation are the most frequently used methods. Physical activation is usually performed using carbon dioxide or steam [18,19]. Potassium compounds, such as potassium hydroxide, potassium carbonate, potassium oxalate [20,21,22,23] or zinc chloride [24,25], are often used for the chemical activation of carbon. Ludwinowicz and Jaroniec [21] obtained carbon spheres using potassium oxalate as an activator with a high surface area equal to 2130 m^2^/g. Cesano et al. [26] produced large (diameter of 100–300 µm) polymer waste-derived microporous carbon microspheres starting from a poly(4-ethylstyrene-co-divinylbenzene) precursor, which was infiltrated by ZnCl_2_. The incorporation of ZnCl_2_ phase into the microporous scaffold of the polymer prevented the collapse of the pore structure and played a role of an activating agent during the pyrolysis carried out at 800 °C. As a result, an extended mesoporosity of the carbon material was obtained. The same groups [27] applied two activating agents, ZnCl_2_ and KOH, to obtain carbon material from the same precursor. The authors concluded that KOH favored the development of microporosity and the surface area, while ZnCl_2_ promoted the mesoporous character of the carbon spheres. Chang et al. [28] used a cheap carbon source, glucose, to produce ZnCl_2_-activated porous carbon spheres with a high surface area and superior mesoporous structure. A very high specific surface area of 2437 m^2^/g was attained by Wang et al. [24], in the case of the mesoporous activated carbon spheres derived from the resorcinol-formaldehyde resin using zinc chloride as an activator.

The usefulness of these materials as carbon dioxide sorbents have been widely proved [9,29,30]. In the paper of Wickramaratne [31], the adsorption value of the CO_2_ at 0 °C and 1 atm for the carbon spheres produced from the resorcinol-formaldehyde resin reached 8.05 mmol/g. Kim et al. [32] synthesized superior CO_2_ adsorbents from the resorcinol-formaldehyde resin via a sol–gel reaction and calcination, and additionally activated it with hot NH_3_ and CO_2_. Due to the ultrahigh specific surface area (4079 m^2^/g) and ultra-microporous structure, the N-doped nanocomposite materials (containing 2.8 wt.% of N) presented a high adsorption capacity: 7.14 and 4.54 mmol/g measured at 0 and 25 °C and 1 atm, respectively. Wang et al. [29] prepared a highly effective (4.83 mmol/g of adsorbed CO_2_ at 25 °C and 1 atm) microporous CS via the direct carbonization of resorcinol-formaldehyde resin spheres mixed with a potassium activating agent. In turn, Khodabakhshi et al. [33] proposed an ecofriendly, rapid and activation-free CVD method to synthesize ultra-microporous CS from pyromellitic acid. The adsorption capacity calculated for this group of green carbon spheres reached 4.0 and 2.9 mmol/g at 0 °C and 25 °C and 1 atm, respectively. 

The application of other carbon materials as CO_2_ sorbents has been recently widely investigated. Activated carbon derived from biomasses, e.g., rice husk [34] or wheat flour [35], exhibit a CO_2_ uptake equal to 6.24 and 5.70 mmol/g, respectively, at 0 °C and 1 bar. Acid-treated multiwalled carbon nanotubes expressed CO_2_ uptake at 2.53 mmol/g at 0 °C and 1 bar [36]. Another promising group of carbon materials for CO_2_ adsorption are carbon fibers. The CO_2_ adsorption of these materials derived from polyacrylonitrile can reach 3.16 mmol/g at 25 °C and 1 bar [37]. Politakos et al. [38] tested the CO_2_ adsorption capacity on graphene-based monolithic nanostructures reaching 2.1 mmol/g at 25 °C and 1 atm. The functionalization of graphene sheets with, i.e., ionic liquids, highly increases the CO_2_ adsorption to 8.5 mmol/g (at 25 °C and 1 bar) [39]. 

In this work, we present the preparation of ZnO/carbon sphere composites using a resorcinol-formaldehyde resin as a carbon source and zinc chloride as a zinc oxide source in a solvothermal reactor heated with microwaves. The influence of the activation with potassium oxalate and the modification with zinc chloride on the physicochemical properties of the obtained materials and CO_2_ adsorption capacity is investigated and discussed. The research on the new composites consisting of carbon spheres and photoactive compounds is justified due to the possibility of obtaining materials allowing for the simultaneous adsorption of carbon dioxide and, due to the presence of semiconducting zinc oxide, its processing into useful products in the process of photocatalytic reduction. 

## 2. Materials and Methods

### 2.1. Materials Preparation

To prepare the reference sample (denoted as RF), 0.6 g of resorcinol was dissolved in an aqueous alcohol solution composed of 60 mL of distilled water and 24 mL of ethanol. Optionally, potassium oxalate was introduced as an activating agent to the mixture and the solution was stirred until the potassium oxalate was completely dissolved. The weight ratio of potassium to carbon equaled 7:1. The activated material was denoted as RF7/1. To adjust the pH~9, the appropriate amount of ammonium hydroxide (25 wt.%) was dropped into the beaker. Finally, 0.9 mL of formaldehyde (37 wt.%) was added to the solution, and the solution was mixed using a magnetic stirrer at ambient conditions to facilitate a polycondensation reaction. After 24 h, the mixture was placed into a Teflon vessel and transferred to a microwave reactor (ERTEC MAGNUM II, Poland). The processes were conducted for 15 min under a pressure of 20 bar. Next, the samples were dried for 48 h at 80 °C and then carbonized in a high-temperature furnace (HST 12/400 Carbolite) under an argon atmosphere, with the temperature rising from 20 to 350 °C with a heating rate of 1 °C/min, and a holding time of 2 h with the temperature increasing from 350 to 700 °C with a heating rate of 1 °C/min. When a temperature of 700 °C was reached, carbonization was continued for 2 h. Afterwards, the samples were cooled to room temperature under an argon atmosphere. The final products were washed with distilled water and dried for 48 h at 80 °C in the laboratory dryer. 

To the modification of the obtained carbon materials, zinc chloride was added. The weight ratio of carbon to zinc equaled 10:1 or 2:1. The obtaining procedure was almost the same as in the case of the reference materials, except that the zinc chloride was added during the polycondensation reaction or after microwave treatment. In the first case, it means that ZnCl_2_ was added after the addition of resorcinol (the samples denoted as RF + ZCl_10/1, RF + ZCl_2/1) or the optional resorcinol and potassium oxalate (the samples denoted as RF7/1 + ZCl_10/1, RF7/1 + ZCl_2/1). In the second case, a proper amount of ZnCl_2_ was dissolved in 50 mL of distilled water, and the solution was added to the materials after microwave treatment, and the pH value was adjusted to ~9 using ammonium hydroxide (25 wt.%). The solution was mixed with a magnetic stirrer for 24 h and then dried and carbonized, as previously mentioned (the samples without potassium oxalate are denoted as RF + ZCl_10/1(1), RF + ZCl_2/1(1), and the sample with potassium oxalate is denoted as RF7/1 + ZCl_10/1(1)). 

### 2.2. Materials Characterization

The morphology of the obtained materials was investigated using a SU8020 Ultra-high Resolution Field Emission Scanning Electron Microscope (Hitachi Ltd., Chiyoda, Tokyo, Japan). The phase composition was investigated with X-ray diffraction using Cu Kα radiation (λCu Kα = 0.1540 nm) on an Empyrean (Panalytical, Malvern, UK). Phase identification was performed using HighScore+ and the ICDD PDF-4+ 2015 database. 

Thermogravimetric measurements were conducted to study the thermal stability of the tested materials within the temperature range of 25–950 °C. In addition, thermal analysis was carried out to obtain data on the decomposition processes, thermal stability and temperature of the phase transformations of the prepared materials. Thermal analysis was conducted using STA 449 C thermobalance (Netzsch Company, Selb, Germany). The samples (ca. 10 mg) were placed in the TGA sample pan and heated from an ambient temperature to reach 950 °C, at the heating rate of 10 °C/min under the airflow of 30 cm^3^/min.

The characterization of the porosity was performed using N_2_ adsorption/desorption on a QUADRASORB evoTM Gas Sorption automatic system (Quantachrome Instruments, Anton Paar Group AB) at −196 °C. The Brunauer–Emmett–Teller (BET) equation was used to determine the surface area (S_BET_), and S_BET_ was determined to be in the relative pressure range of 0.05–0.3. The total pore volume, TPV, was calculated from the volume of nitrogen held at the highest relative pressure (*p*/*p*_0_ = 0.99). The volume of micropores, V_m_, with dimensions less than 2 nm was calculated by integrating the pore volume distribution function using the DFT method; the mesopore volume, V_meso_, with dimensions from 2 to 50 nm was calculated from the difference of the total pore volume, TPV, and the volume of micropores, V_m_. Before each adsorption experiment, the samples were outgassed at 250 °C under a vacuum of 1 × 10^−5^ mbar for 12 h using a MasterPrep multi-zone flow/vacuum degasser from Quantachrome Instruments to remove adsorbed species that could intervene in the adsorption processes. Carbon dioxide adsorption isotherms at 0 and 25 °C were measured using the same Quadrasorb™ automatic system (Quantachrome Instruments, Boynton Beach, FL, USA) in the pressure range between 0.01 and 0.98 bar. The Pore Size Distribution (PSD) of the samples was calculated from CO_2_ sorption isotherms at 0 °C using the NLDFT model. The volume of ultra-micropores, V_s_, with dimensions less than 1.0 nm (<1 nm) was determined from the CO_2_ adsorption isotherm at 0 °C, and calculated by integrating the pore volume distribution function using the NLDFT method. 

CO_2_ adsorption–desorption measurements were additionally carried out using Netzsch STA 449 C thermobalance, based on weight gain and loss during sorption at 40 °C and a desorption process at 105 °C. The CO_2_ adsorption experiment was reported in detail in our previous works [10,40], so the procedure is only summarized in the present paper. First, the sample was dried at 105 °C in pure N_2_ for 60 min to remove moisture and/or CO_2_ from the sample. Then, the temperature was cooled to the adsorption temperature and kept for 60 min. Next, the gas was switched from N_2_ to CO_2_, and the CO_2_ sorption process started. After the sorption, the temperature increased to 105 °C, and pure CO_2_ was switched off; N_2_ was introduced into the sample chamber for carbon dioxide desorption. 

To assess the material stability as a CO_2_ sorbent, cyclic adsorption–desorption tests were performed. The experiments were carried out for the sample with the largest adsorption capacity among all the prepared samples. The adsorption experiments were carried out at 40 °C under pure CO_2_ flow for 60 min, and then CO_2_ was desorbed at 105 °C under pure N_2_ flow for 60 min. The procedure was conducted for 25 cycles to test the stability and adsorptive repeatability of the nanomaterials. The CO_2_ sorption capacity (mmol CO_2_/g of sorbent) was calculated according to the mass change of the sample after each adsorption–desorption run.

## 3. Results and Discussion

**Figure 1:**
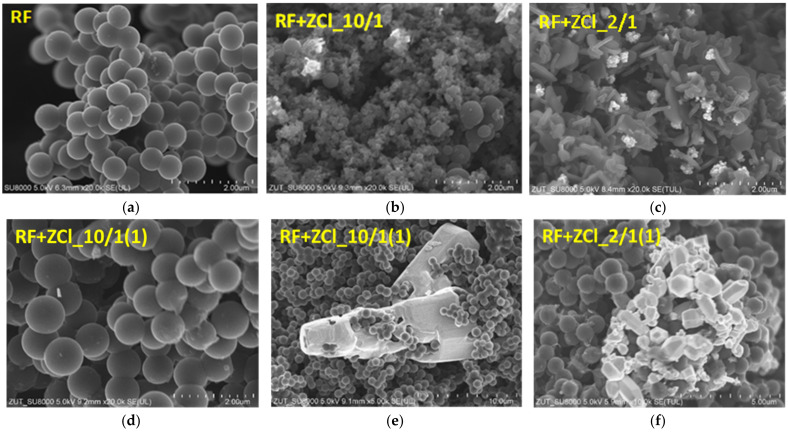
SEM images of the reference sample RF (**a**) and samples modified using zinc chloride (**b**–**f**).

The morphology of the reference materials was studied using scanning electron microscopy and compared with the morphology of the materials modified using zinc chloride, to determine the effect of the modifier addition. Scanning electron images are presented in Figure 1. For the non-activated sample (without an addition of potassium oxalate), carbon spheres with a regular shape, smooth surface, with no inclusions or impurities and with a mean diameter of about 600 nm can be observed (Figure 1a). The detailed characteristics of carbon spheres obtained under different experimental conditions has been described in previous work [9,10,11]. 

SEM images of the non-activated and modified using zinc chloride samples are presented in Figure 1b–f. It was observed that the addition of modifier during the polycondensation reaction disrupted the process of carbon sphere synthesis, regardless of the amount of added zinc chloride. In the case of the RF + ZCl_10/1 sample (Figure 1b), carbon spheres with non-homogeneous diameters can be observed. Very fine carbon spheres with diameters of about 100 nm and larger ones with diameters of 500 nm were identified. Zinc oxide crystals were also observed in the carbon matrix. The addition of a higher amount of zinc chloride during the polycondensation step (RF + ZCl_2/1 sample) resulted in the complete destruction of the spherical structures of carbon, which can be observed in Figure 1c, where only a flake structure can be noticed. 

For the reason mentioned above, the preparation of the modified carbon spheres was slightly changed and zinc chloride was added not during the polycondensation step, but after microwave treatment. It was found that in both the cases involving RF + ZCl_10/1(1) and RF + ZCl_2/1(1), the spherical shape of carbon was preserved. The diameters of the carbon spheres were about 700–900 nm for both the RF + ZCl_10/1(1) (Figure 1d) and RF + ZCl_2/1(1) (Figure 1f) materials. Additionally, apart from spherical carbon, the large hexagonal zinc oxide crystals with a size above 20 µm appeared in the material (Figure 1e,f). 

It is known that the spherical carbon spheres can be obtained without heat treatment, hence the shape of the carbon materials is formed during the polycondensation reaction [41]. During a standard activation of carbon material with zinc chloride, the liquid chemical is intercalated into the carbon matrix to produce pores at a temperature above the melting point of the chemical agent [42]. In the case of the procedure applied in our experiments for the samples RF + ZnCl_10/1(1) and RF + ZnCl_2/1(1), the addition of zinc chloride to the already formed carbon spheres can inhibit the intercalation phenomenon (zinc chloride cannot penetrate the forming spheres), and therefore it can simultaneously lead to the preservation of the spherical shape and the formation of large zinc oxide crystals on the carbon surface, but not to the development of the porosity of the material, as is possible during polymerization. The attached SEM images of such samples presented in Figure 1d–f show that the spherical shape of the carbon material is preserved in this case. Moreover, we can observe very well-crystallised hexagonal ZnO structures in these samples (Figure 1e,f).

**Figure 2:**
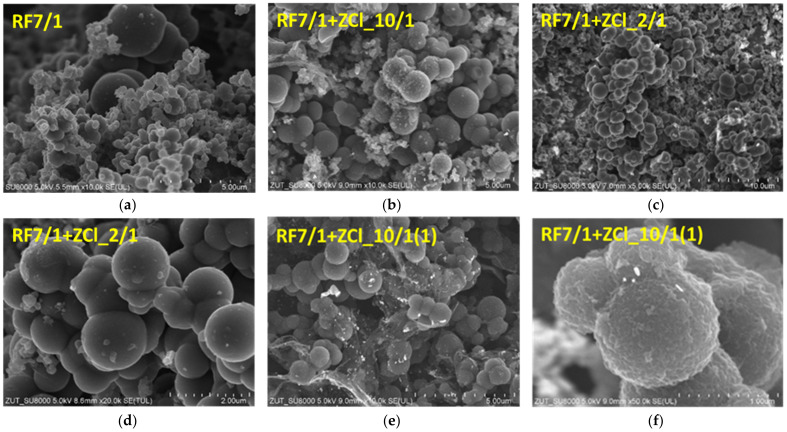
SEM images of the materials activated with potassium oxalate (**a**) and activated materials modified using zinc chloride (**b**–**f**).

The morphology of the reference material activated with potassium oxalate is presented in Figure 2a. It can be stated that the addition of an activator caused significant changes in the sphere’s morphology. Due to the chemical activation, a differentiation of the size of the spheres occurred—we can observe both large spheres with a diameter of about 1 μm, but also much smaller spheres with a diameter below 500 nm. Additionally, the surface of the spheres became less smooth, and some excrescences and small flakes were visible on the surface of the spheres. The images presented in Figure 2b show the morphology of the sample RF7/1 + ZCl_10/1, to which ZnCl_2_ was added after the addition of resorcinol and potassium oxalate. In this sample, we can see large spheres of about 1 μm and smaller structures below 500 nm, forming agglomerates. The increase in the amount of ZnCl_2_ added during the synthesis caused the inhibition of the chemical activation of the sample RF7/1 + ZCl_2/1, which can be observed in Figure 2c,d. We can see the smooth surface of the spheres. The shape of the spheres can suggest that the activation occurred to a slight degree, and that the addition of ZnCl_2_ did not affect the morphology of the spheres in a meaningful way. In the samples without adding K_2_C_2_O_4_, an increase in ZnCl_2_ concentration causes the destruction of the spheres, and flake carbon structures can be seen in Figure 1b,c. The explanation of the inhibiting effect of the simultaneous addition of both activating agents (K_2_C_2_O_4_ and ZnCl_2_) on the activation process is discussed further in the present paper, using the conclusions based on XRD measurements. The SEM images of the activated sample, in which zinc chloride was added to the precursor after the synthesis in the microwave-assisted solvothermal reactor, but before the carbonization, are shown in Figure 2e,f. In this case, some graphene-like or graphite-like structures can be clearly visible between the carbon spheres. Additionally, it can be seen that the surface of the spheres is composed of overlapping carbon flakes (Figure 2f). The presence of the ZnO crystallites can be observed in the sample, both on the surface of the carbon spheres and on graphene-like structures. Contrary to the non-activated samples, large crystalline ZnO structures were not noticed. 

**Figure 3:**
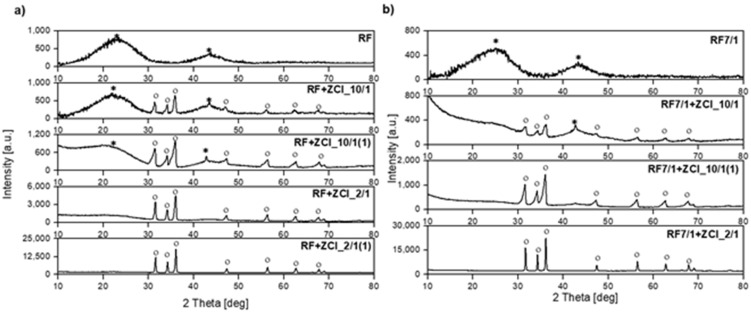
Diffraction patterns of (**a**) the reference material RF and non-activated materials modified with zinc chloride, (**b**) reference material RF7/1 and activated with potassium oxalate and modified with zinc chloride materials. Reflections attributed to carbon (ICDD 00-041-1487) are marked as *, reflections attributed to hexagonal zinc oxide (ICDD 01-079-0208) are marked as o.

The phase composition of the received samples was characterized using the X-ray diffraction method. The diffraction patterns of the non-activated and K_2_C_2_O_4_-activated samples are presented in Figure 3a,b, respectively. 

For the non-activated reference sample (RF), the broad diffraction peaks centered around 23° and 43° were noticed and corresponded to (002) and (100) planes, respectively [43,44]. The broadening of carbon peaks suggests a low degree of graphitization and the possible presence of amorphous carbon [45,46,47]. For all non-activated and modified materials, the same phase composition was determined, and apart from carbon, the peaks corresponding to zinc oxide were identified. As expected, the intensity of zinc oxide peaks for the samples with a higher zinc content (a carbon-to-zinc mass ratio equal to 2:1) was more intensive than in the case of the samples with a lower zinc content (a carbon-to-zinc mass ratio equal to 10:1). What is important, regardless of the C-to-Zn mass ratio, is that a higher intensity of ZnO peaks was observed for the materials to which zinc chloride was added after microwave treatment (the samples RF + ZCl_10/1(1) and RF + ZCl_2/1(1)). This is because the addition of ZnCl_2_ after microwave treatment inhibited the penetration of the carbon matrix by Zn ions. Consequently, a higher amount of ZnO was formed on the surface of the carbon spheres in these materials.

In the samples prepared without the addition of potassium oxalate, the asymmetry of the ZnO reflections was observed. The reason for the asymmetry of ZnO reflections is the phenomenon of “specimen transparency error”. Carbon has a low X-ray absorption coefficient. Therefore, the beam incident on the sample penetrates to a great depth, and diffraction occurs not only on the surface but also in a large sample depth range. Furthermore, a low carbon absorption coefficient causes the asymmetric blurring of diffraction reflections towards lower angles [48]. The mass absorption coefficient (MAC) for the CuKα_1_ line equals 50.34 cm^2^/g for ZnO and 4.30 cm^2^/g for carbon, respectively. Then, under the same experimental conditions, the depth of penetration of the radiation beams into the samples is 56 μm for ZnO and 1830 μm for carbon.

The shape and position of the carbon peaks in all the samples were similar to the reference sample. The broadened peaks for the samples RF + ZCl_10/1(1) and RF + ZCl_2/1(1) were hardly visible due to the high intensity of the ZnO peaks determined for these materials. Additionally, it is worth noting that for the samples RF + ZCl_10/1 and RF + ZCl_10/1(1), a sharper peak at 2 theta equaled 43.5°, for which a greater intensity was observed and assigned to the carbon (100) plane. Its presence can suggest the higher graphitization of carbon material [43,44]. 

In the case of the reference sample activated with potassium oxalate (RF7/1), the width of the peak located at 23° increased and the peak maximum shifted towards the angle of 25°. This means that the degree of carbon graphitization may decrease in the activation process. For the activated and modified samples, the peaks corresponding to zinc oxide were also identified, and similar to the case of the non-activated samples, the asymmetry of these reflections was observed for the samples where the carbon-to-zinc mass ratio was 10:1.

**Figure 4:**
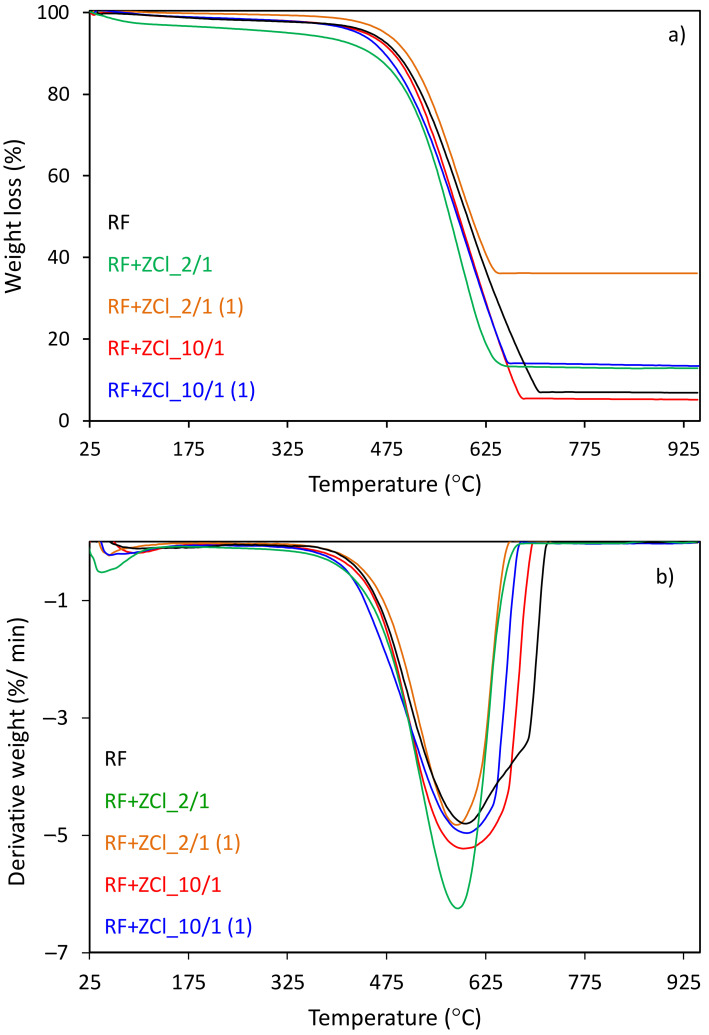
(**a**) TG and (**b**) DTG curves of the unmodified RF and carbon spheres modified with different amounts of zinc chloride salt as a ZnO precursor before and after microwave treatment.

Figure 4a,b shows the thermal analyses for the group of carbon spheres modified with zinc chloride salt as a ZnO precursor, before and after microwave treatment, determined by the thermogravimetry (TG) and differential thermogravimetry (DTG) analyses. According to these curves, the first peaks appear at the temperature scope between 40 and 150 °C, which could be interpreted as an elimination of H_2_O formed from the condensation of –OH groups. The total weight loss of about 91 %, in the case of the unmodified sample (RF), was observed between 330 and 730 °C, corresponding to the differential thermogravimetric (DTG) profile with maximum temperatures at 600 and 680 °C. The first decomposition reaction, which occurs at a lower temperature, could be attributed to breaking C–O bonds. In contrast, the second decomposition reaction occurring at a higher temperature is assigned to C–H bond breakage in favor of C–C bond formation and is highly likely responsible for the desorption of residual organic compounds [49].

The results also show that the more zinc chloride contained in the sample, the lower the initial and final thermal decomposition temperatures can be observed. As can be seen from the recorded curves, the heating of the samples to 950 °C resulted in a complete thermal decomposition of the compound above 700 °C (TG and DTG curves), which was accompanied by decomposition peaks in the DTG profile between 360–700 °C and between 280–680 °C with a maximum at 600 and 580 °C for the samples RF + ZCl_10/1 and RF + ZCl_2/1, respectively. The total weight loss for these samples reached 95 and 87%, respectively. For the RF + ZCl_2/1(1) sample, we did not observe any significant differences in the thermal decomposition compared to the sample when the zinc chloride was added before the polycondensation process. The total weight loss for the RF + ZCl_2/1(1) sample reached 65%.

**Figure 5:**
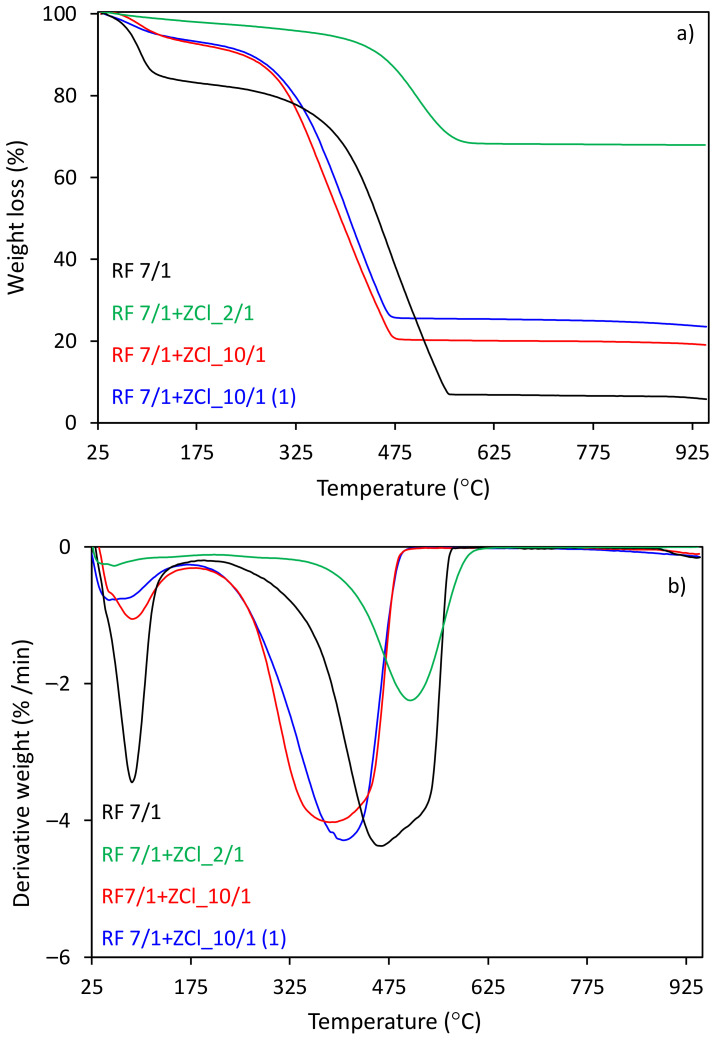
(**a**) TG and (**b**) DTG curves of the unmodified RF7/1 and activated carbon spheres modified with different amounts of zinc chloride before and after microwave treatment.

As shown in Figure 5, the utilization of potassium oxalate as an activator results in a decrease in the thermal stability of carbon spheres. The total weight loss for the sample with the addition of potassium oxalate reached ca. 81% and 77%, which was accompanied by the decomposition peaks (DTG curves) between 174–390 °C and 200–460 °C, reaching a maximum amount with the DTG peak at 395 °C for RF7/1 + ZCl_10/1 and 410 °C for RF7/1 + ZCl_10/1(1), respectively. However, a different situation was observed in the case of carbon modified with the largest amount of zinc chloride salt as a ZnO precursor (RF7/1 + ZCl_2/1). The total weight loss for this sample reached 32%, whereas decomposition occurred in the range of 350–620 °C, reaching the maximum rate of decomposition at 530 °C (DTG profile). We can observe that the initial, final and maximum thermal decomposition temperatures are similar to the same material before activation with potassium oxalate, which indicates that the activation process in the case of material modification with the largest amount of zinc chloride did not take place. 

**Figure 6:**
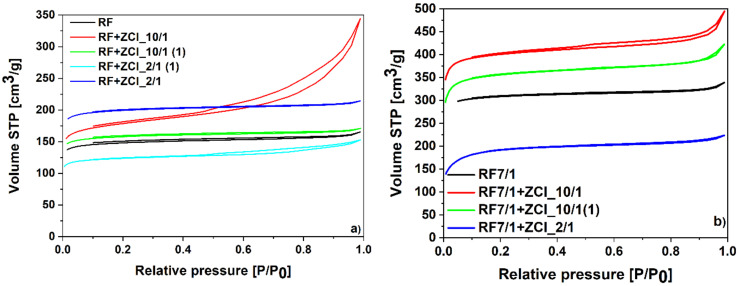
N_2_ sorption isotherms of the (**a**) nonactivated and (**b**) activated samples modified with ZnCl_2_.

The specific surface area (S_BET_), the total pore volume (TPV), the volume of micropores (V_m_ < 2 nm) and the volume of mesopores (V_meso_) were determined based on the low-temperature (−196 °C) nitrogen adsorption isotherms presented in Figure 6a, for non-activated materials, and in Figure 6b for materials activated with potassium oxalate. The values are listed in Table 1.

It is clearly visible that the non-activated materials (without potassium oxalate) modified using zinc chloride added during the polycondensation step, possessed a higher specific surface area and total pore volume than the reference sample (RF). The specific surface area for RF material equaled 455 m^2^/g, whereas for RF + ZCl_10/1 and RF + ZCl_2/1, it equaled 561 m^2^/g and 612 m^2^/g, respectively. The value of the total pore volume increased from 0.26 cm^3^/g for the RF sample up to 0.53 cm^3^/g for RF + ZCl_10/1 and 0.33 cm^3^/g for RF + ZCl_2/1 materials. In contrast to this, the materials modified using zinc chloride, but after microwave treatment, did not have such a developed specific surface area. The S_BET_ for RF + ZCl_10/1(1) equaled 485 m^2^/g and it was similar to the value obtained for the reference material, whereas the S_BET_ for RF + ZCl_2/1(1) was even lower and equaled only 381 m^2^/g. Moreover, the addition of the zinc chloride after microwave treatment did not increase the total pore volume compared to the reference material. The above-presented results correlate well with those obtained using scanning electron microscopy and confirm that the addition of zinc chloride after microwave treatment causes the spherical shape of carbon to be preserved, but simultaneously the production of the pores is hindered or prevented.

The activation of the carbon material using potassium oxalate increased the specific surface area value from 455 m^2^/g for the reference sample, RF, to 954 m^2^/g for the sample RF7/1. When the modifications of the materials with a lower content of ZnCl_2_ were applied, a further increase in the specific surface area and total pore volume values was noticed, regardless of the application method. However, when the ZnCl_2_ concentration was too high for the sample RF7/1 + ZCl_2/1, a substantial decrease in S_BET_ and TPV values was noticed. 

**Figure 7:**
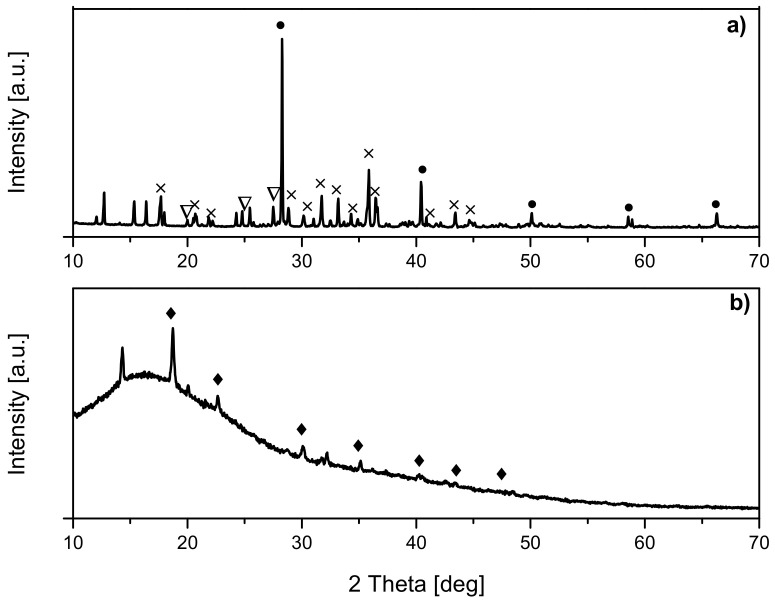
Diffraction patterns of the sample RF7/1 + ZCl_2/1 (**a**) before carbonization, and (**b**) before carbonization and after washing with distilled water. Reflections attributed to zinc oxalate (ICDD 00-014-0740) are marked as ◆, potassium chloride (ICDD 01-075-0296) are marked as •, potassium oxalate (ICDD 00-022-1232) are marked as ×, zinc hydroxide (ICDD 01-089-0138) are marked as ∇.

In order to explain this phenomenon, the sample RF7/1 + ZnCl_2/1 was obtained, but omitting the carbonization step. Therefore, the received material was investigated using the X-ray diffraction method, and the diffraction pattern is presented in Figure 7a. The following phases were detected in this sample: potassium chloride, potassium oxalate, and zinc hydroxide, according to the obtained results. Apart from the mentioned phases, other reflections were also observed, but the high intensity of potassium chloride made their identification impossible. Therefore, the same sample was washed with distilled water and tested again using the diffraction method. Only zinc oxalate was detected on the diffraction pattern presented in Figure 7b.

Considering the results described above, it can be stated that the modification of carbon spheres simultaneously with potassium oxalate (mass ratio of K:C = 7:1) and with zinc chloride (mass ratio of C:Zn = 2:1) did not result in the activation of carbon material. During the synthesis of the composite, some parts of the potassium ions from K_2_C_2_O_4_ reacted with chloride ions forming potassium chloride. One can suppose that the potassium used to form potassium chloride was not involved in the sample activation. This hypothesis is supported by SEM observations, which showed that the material was composed mainly of carbon spheres. Some larger ZnO crystallites (aggregates) and single or agglomerated small ZnO aggregates can be observed on the surface of carbon material. Zinc oxide covering the surface of the spheres can partially block access to the pores in the carbon matrix. 

These two phenomena, the lack of activation and blocking of pores by ZnO, can inhibit CO_2_ adsorption by the sample, which had the lowest values of specific surface area and CO_2_ adsorption among all the activated samples. In addition, thermogravimetric studies also confirmed the lack of the activating effect of the carbon material with ZnCl_2_. 

The nitrogen sorption isotherms of the non-activated materials modified with ZnCl_2_ are presented in Figure 6a. The investigation of the textural properties of samples using N_2_ sorption isotherms showed that nonactivated reference material, RF, is microporous (isotherm of type I according to the IUPAC classification). Considering the samples with a lower concentration of ZnCl_2_, the application method strongly affects the textural properties of final carbon materials. When ZnCl_2_ is added before polycondensation, a higher content of mesopores in the material was obtained (V_meso_ value increased from 0.04 cm^3^/g for the reference sample RF to 0.25 cm^3^/g for the sample RF + ZCl_10/1). Moreover, a H3 hysteresis loop for this material was observed. The development of mesopore content was not observed for the sample RF + ZCl_10/1(1). The addition of ZnCl_2_ after heat treatment of the resin in the reactor resulted in preserving the microporosity of the carbon materials. The N_2_ isotherms of the carbon materials with the higher content of ZnCl_2_ were of type I characteristic for the microporous material. The highest content of micropores was achieved for the sample RF + ZCl_2/1, where ZnCl_2_ was added before microwave treatment. When ZnCl_2_ was added after microwave treatment, a lower content of N_2_ was adsorbed (sample RF + ZCl_2/1(1)) and a H4 hysteresis loop was observed. It is likely that carbon material after microwave treatment is more stable and less prone to modification with ZnCl_2_. 

As shown in Figure 6b, the unmodified activated carbon material RF7/1 expresses the nitrogen isotherm of type I characteristic for the microporous materials. Samples modified with a lower ZnCl_2_ concentration, regardless of the application method, adsorbed higher values of N_2_ than the unmodified sample RF7/1. The isotherms obtained for these samples were mixed type I and type II, indicating a high mesopore content. Moreover, for the material RF7/1 + ZCl_10/1, a H4 hysteresis loop was noticed. This observation is in accordance with the previous statement that the application method of ZnCl_2_ affects the surface of carbon materials. The addition of ZnCl_2_ after the reactor treatment (sample RF7/1 + ZCl_10/1(1)) increased the N_2_ adsorbed volume compared to the RF7/1 sample, but no hysteresis loop was observed. The N_2_ adsorption isotherm obtained for the material with the highest Zn content, RF7/1 + ZCl_2/1, is a type I characteristic for the microporous materials. Although both activating agents were present in this sample, the decrease in the total pore volume compared to the RF7/1 sample was noticed. This result occurred due to potassium chloride and zinc oxalate, which could not interact effectively with the carbon matrix. 

**Figure 8:**
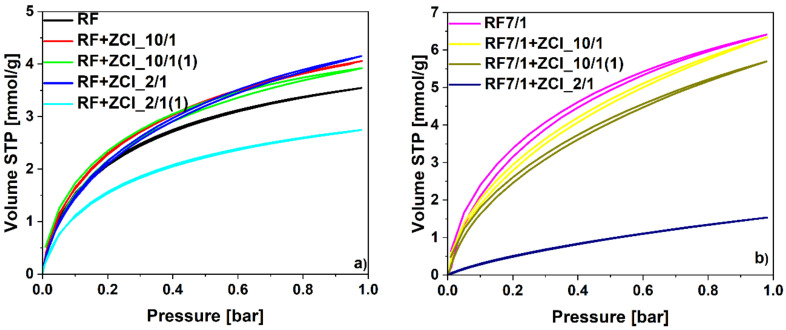
CO_2_ sorption isotherms at 0 °C of (**a**) non-activated and (**b**) activated materials modified with zinc chloride.

The influence of the carbon material’s modification on CO_2_ uptake was also investigated based on CO_2_ adsorption isotherms presented in Figure 8. The calculated values of CO_2_ adsorption capacity at 0 and 25 °C are summarized in Table 1. For the non-activated materials (without potassium oxalate), the highest value of the CO_2_ adsorption at 0 °C (Figure 8a) was noticed for the sample for which the concentration of Zn was the highest and ZnCl_2_ was added before polycondensation (sample RF + ZCl_2/1). On the other hand, for the sample RF + ZCl_2/1(1), for which ZnCl_2_ was added after microwave treatment, a strong decrease in the CO_2_ adsorption value was noticed. For the samples modified with the lower ZnCl_2_ content, regardless of the application method, higher CO_2_ adsorption values were achieved than for the reference material RF. The same dependence was observed in the case of CO_2_ adsorption studied at 25 °C. 

Due to the development of microporosity, the chemical activation of the carbon materials strongly improved the CO_2_ adsorption values, from 3.25 mmol/g for the RF sample to 6.41 for the RF7/1 sample. For the modified samples activated using potassium oxalate, RF7/1 + ZCl_10/1 and RF7/1 + ZCl_10/1(1), a decrease in the CO_2_ adsorption ability was noticed, compared to the values received for the activated reference material RF7/1. The additional application of ZnCl_2_, which is also a well-known activating agent, did not improve the value of the CO_2_ adsorption capacity. When the ZnCl_2_ was added before the polycondensation the CO_2_, adsorption was not diminished as much as when the ZnCl_2_ was added after high-pressure treatment in the solvothermal reactor. For the sample RF7/1 + ZCl_2/1, the application of the higher content of ZnCl_2_ caused a strong decrease in S_BET_ and CO_2_ adsorption values, due to the creation of potassium chloride and zinc oxalate, which impeded the activating potential of the activators. 

**Figure 9:**
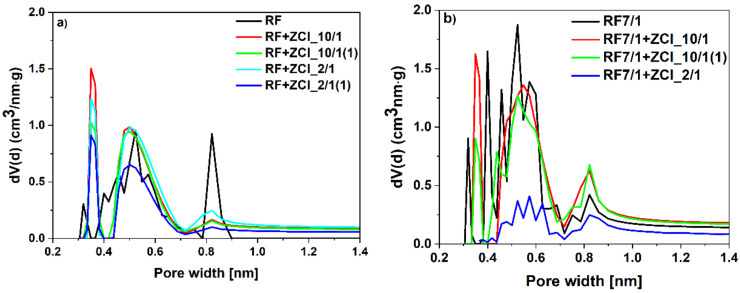
PSD of the (**a**) non-activated and (**b**) activated samples modified with zinc chloride.

For the materials without potassium oxalate, modification with ZnCl_2_ resulted in increased CO_2_ adsorption at 0 °C. The pore size distributions of these samples are presented in Figure 9a. For the materials modified with ZnCl_2_, a higher content of pores that were 0.35 nm in size was detected, compared to the reference sample, RF. Moreover, the development of the pores that were 0.55 nm in size was noticed. This could result in an increase in CO_2_ adsorption values. The lowest contribution of pores that were 0.35 nm and 0.55 nm in size was observed for the sample RF + ZCl_2/1(1). Consequently, this sample expressed the lowest CO_2_ adsorption values. The modification of the carbon materials with ZnCl_2_ resulted in a decrease in the content of pores that were 0.85 nm in size. Nevertheless, for efficient CO_2_ adsorption, pores below 0.7 nm in size are essential, thus this phenomenon did not diminished the CO_2_ adsorption values [20]. 

The same effect is observed in Figure 9b, where pore size distributions of the samples activated with potassium oxalate and modified with zinc chloride are presented. In these samples, we have the highest proportions of micropores below 1 nm. Unmodified sample RF7/1 possessed the highest content of pores that were 0.4 nm and 0.5 nm in size. The modification of carbon materials with ZnCl_2_, in samples RF7/1 + ZCl_10/1 and RF7/1 + ZCl_10/1(1), resulted in the shift in the size of the smallest pores towards lower values (from 0.4 nm to 0.35 nm in size), and the decrease in the content of pores that were 0.5 nm in size. In the sample RF7/1 + ZCl_2/1, the contribution of pores below 1nm was the lowest, and the activation was not performed.

**Figure 10:**
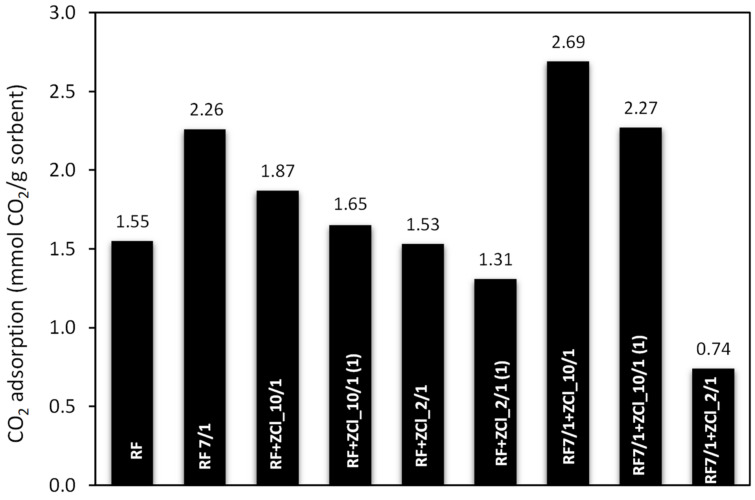
A comparison of the CO_2_ adsorption capacity at 40 °C of the samples used in this study.

For comparison purposes, the CO_2_ uptake on produced materials was also investigated using thermogravimetry at 40 °C. Such a high adsorption temperature is closer to natural conditions. The adsorption experiments for all samples are shown in Figure 10. The CO_2_ adsorption capacity for unmodified material reached 1.55 mmol/g, whereas for the sample after activation, the CO_2_ adsorption capacity was 2.26 mmol/g. The porous structure of the material and the specific surface area play significant roles in the adsorption processes. The specific surface area (S_BET)_ for the sample after activation increased by nearly 500 m^2^/g, while the total pore volume (TPV), the volume of ultra-micropores (V_s_) as well as the volume of micropores (V_m_) increased twice, compared to the material untreated with potassium oxalate. The activation process plays a crucial role in forming new micro-, meso- and ultra-micropores, providing a sustaining increase in the surface area and pore volume. Our previous works thoroughly explained the effect of potassium oxalate as an activator [10,50].

For the RF + ZCl_10/1 sample, the CO_2_ adsorption capacity was 1.87 mmol/g, but in the case of material RF + ZnCl___10/1(1), the CO_2_ sorption capacity decreased to 1.65 mmol/g. A similar correlation can be observed in the case of the materials RF + ZnCl___2/1 and RF + ZCl_2/1(1); the CO_2_ sorption capacity reached 1.53 mmol/g and decreased to 1.31 mmol/g, accordingly. It corresponds to a ca. 12 and 14% decrease compared to the carbon spheres RF + ZCl_10/1 and RF + ZCl___2/1. The results show that the addition of the zinc chloride after microwave treatment leads to the formation of carbon spheres with a less developed specific surface area and poor porous properties, compared to the carbons to which zinc chloride was added before polycondensation. The specific surface area for RF + ZCl_10/1(1) was 485 m^2^/g and decreased to 381 m^2^/g for RF + ZCl_2/1(1). It corresponds to a ca. 26 and 40% decrease compared to the carbon spheres RF + ZCl_10/1 and RF + ZCl_2/1, respectively. Furthermore, considering the textural parameters (Table 1), it can be observed that the adsorption capacity for CO_2_ in the case of samples RF + ZCl_10/1(1) and RF + ZCl_2/1(1), where the ZnCl_2_ was added after microwave treatment, decreases with a decreasing total pore volume and the volume of micropores compared to the samples to which the ZnCl_2_ was added during polycondensation. Adsorption capacity changes significantly after the activation process, in the case of RF7/1 + ZCl_10/1 and RF7/1 + ZCl_10/1(1) materials, and reached 1.69 and 2.27 mmol/g, and it was 1.44 and 1.38 times higher than the same samples before activation. As expected, the activation process improved both the structural parameters and the specific surface area, S_BET_, increasing from 658 to 1233 m^2^/g and from 485 to 1098 m^2^/g for RF7/1 + ZCl_10/1 and RF7/1 + ZCl_10/1(1), respectively. The adsorption capacity of the carbon modified with a large amount of ZnCl_2_ (RF7/1 + ZCl_2/1) drastically decreased, reaching only 0.74 mmol/g. This fact can be explained by considering the properties of the porous structure of this material after activation with potassium oxalate. The specific surface area, total pore volume and micropore volume did not significantly change after activation, but the volume of the ultramicropores decreased drastically to 0.11 cm^3^/g. 

**Figure 11:**
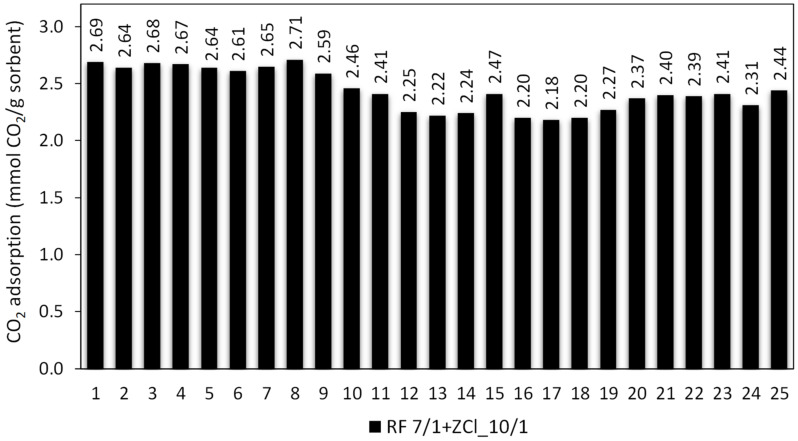
Cyclic stability of CO_2_ adsorption–desorption for the RF7/1 + ZCl_10/1 sample.

The high CO_2_ sorption capacity, regeneration and stability of the sorbent in the cycles are crucial for the practical application of the sorbent, as they are directly related to the cost of the sorption/desorption process. Therefore, the regeneration and recyclability were performed for the RF7/1 + ZCl_10/1 sample characterized by the largest adsorption capacity at 40 °C among all the sorbents in this study. The measured sorption capacities as a function of the cycle number are presented graphically in Figure 11.

The adsorption/desorption cycle experiment revealed that the adsorption performance of RF7/1 + ZCl_10/1 was reasonably stable, without a noticeable degradation in the adsorption capacity of CO_2_ after the 9th cycle; it decreased only by ca. 3.7% compared to the performance in the first cycle. The CO_2_ adsorption capacity between the 9th and 20th cycle decreased from 2.59 to 2.37 mmol/g, i.e., the decrease was ca. 10.3 and 15.6% compared to the performance in the first cycle. The decrease in CO_2_ uptake can be explained by the fact that carbon dioxide molecules enter all pores in the adsorption cycles, especially ultra-micropores, and after desorption, some CO_2_ molecules could not escape efficiently, most likely in the ultra-micropores, due to the closure effect. On the other hand, starting from the 22nd cycle, RF7/1 + ZCl_10/1 showed a virtually stable adsorption–desorption performance during the remaining consecutive runs. These results show that the prepared carbon spheres exhibit a high CO_2_ adsorption capacity and show fairly good stability and regenerability.

## 4. Conclusions

In summary, the chemical activation of carbon materials with potassium oxalate leads to the development of the specific surface area and structural parameters, particularly ultra-microporosity, which significantly impacts the increasing CO_2_ sorption capacity. For the nonactivated and activated materials, the presence of zinc chloride can increase the ultra-micropore volume of carbon spheres, only if it is added during their synthesis (before high pressure microwave treatment). It can also be concluded that a high amount of zinc chloride salt as a ZnO precursor, added to activated material, leads to a decrease in ultra-micropore and mesopore contribution due to potassium chloride and zinc oxalate creation. The addition of zinc chloride after microwave treatment makes it possible to maintain the spherical shape of the material, but it negatively affects the surface area or the total pore volume.

The highest CO_2_ adsorption capacity was observed for the sample activated with potassium oxalate and modified with zinc chloride and reached 6.34 and 3.92 mmol/g at 0 and 25 °C, respectively. Moreover, this material also showed a relatively high CO_2_ adsorption capacity at 40 °C and, during the multiple adsorption–desorption cycles, exhibited a good regeneration and stable performance during 25 consecutive adsorption–desorption cycles. 

## Figures and Tables

**Table 1:** Textural parameters and CO_2_ sorption capacities for the obtained materials.

		S_BET_	TPV	V_s_ (<1 nm)	V_m_ (<2 nm)	V_meso_	CO_2_ 0 °C	CO_2_ 25 °C
		[m^2^/g]	[cm^3^/g]	[cm^3^/g]	[cm^3^/g]	[cm^3^/g]	[mmol/g]	[mmol/g]
RF		455	0.26	0.19	0.22	0.04	3.25	2.43
RF + ZCl_10/1		561	0.53	0.22	0.28	0.25	4.06	2.74
RF + ZCl_10/1(1)		485	0.26	0.22	0.25	0.01	3.92	2.49
RF + ZCl_2/1		612	0.33	0.24	0.29	0.04	4.15	2.73
RF + ZCl_2/1(1)		381	0.24	0.15	0.18	0.06	2.75	1.89
RF7/1		954	0.53	0.37	0.44	0.07	6.41	4.13
RF7/1 + ZCl_10/1		1233	0.77	0.38	0.56	0.21	6.34	3.92
RF7/1 + ZCl_10/1(1)		1098	0.65	0.35	0.50	0.15	5.70	3.51
RF7/1 + ZCl_2/1		602	0.35	0.11	0.26	0.09	1.54	0.84

S_BET_—specific surface area; TPV—total pore volume; V_s_—the volume of ultra-micropores with a diameter less than 1 nm; V_m_—the volume of micropores with a diameter less than 2 nm; V_meso_—the volume of mesopores with diameter from 2 to 50 nm.

## Data Availability

Not applicable.

## Sample Availability

All samples of the compounds described in the article are available from the authors.
